# Screening male prisoners for depression and anxiety with the PHQ-9 and GAD-7 at NHS Healthcheck: patterns of symptoms and caseness threshold

**DOI:** 10.1186/s12888-021-03453-2

**Published:** 2021-09-09

**Authors:** Elizabeth Butcher, Christopher Packham, Marie Williams, Joanne Miksza, Adarsh Kaul, Kamlesh Khunti, Richard Morriss

**Affiliations:** 1 City Hospital, Nottingham University Hospitals NHS Trust, Nottingham, NG5 1PB UK; 2 Nottinghamshire Healthcare NHS Foundation Trust, Nottingham, NG3 6AA UK; 3 Leicester Real World Evidence Unit, University of Leicester, Leicester General Hospital, Gwendolen Road, Leicester, LE5 4PW UK; 4 Primary Care Diabetes and Vascular Medicine, University of Leicester, Leicester General Hospital, Gwendolen Road, Leicester, LE5 4PW UK; 5 Psychiatry, Institute of Mental Health, University of Nottingham, Nottingham, NG7 2DU UK

**Keywords:** Depression disorders, Anxiety disorders, Prison, Screening, Cardiovascular health, Cluster analysis

## Abstract

**Background:**

Screening for depression and anxiety disorders has been proposed in prison populations but little is known about caseness thresholds on commonly used self-report measures in relation to core symptoms, risk factors and symptom patterns.

**Method:**

A cross-sectional prevalence survey measured depression and anxiety caseness (threshold scores > 10 and > 15 on PHQ-9 and GAD-7 and diagnostic algorithm on PHQ-9) in 1205 male prisoners aged 35–74 years eligible for an NHS Healthcheck from six English prisons. Caseness scores were compared with the presence or absence of daily core symptoms of depression and generalised anxiety disorder (GAD), demographic, prison and cardiovascular risk factors. Cluster analysis was applied to PHQ-9 and GAD-7 items in prisoners scoring > 10 on PHQ-9.

**Results:**

453(37.6%) and 249(20.7%) prisoners scored > 10 and > 15 respectively on PHQ-9; 216 (17.9%) had a depressive episode on the PHQ-9 algorithm; 378(31.4%) and 217(18.0%) scored > 10 and > 15 on GAD-7 respectively. Daily core items for depression were scored in 232(56.2%) and 139(74.3%) prisoners reaching > 10 and > 15 respectively on PHQ-9; daily core anxiety items in 282(74.9%) and 179(96.3%) reaching > 10 and > 15 on GAD-7. Young age, prison and previous high alcohol intake were associated with > 15 on the PHQ-9. Cluster analysis showed a cluster with core symptoms of depression, slowness, restlessness, suicidality, poor concentration, irritability or fear. Altered appetite, poor sleep, lack of energy, guilt or worthlessness belonged to other clusters and may not be indicative of depression.

**Conclusions:**

In male prisoners > 35 years, a score of > 10 on the PHQ-9 over diagnoses depressive episodes but a score of > 10 on the GAD-7 may detect cases of GAD more efficiently. Further research utilising standardised psychiatric interviews is required to determine whether the diagnostic algorithm, a higher cut-off on the PHQ-9 or the profile of symptoms on the PHQ-9 and GAD-7 used singly or in combination may be used to screen depressive episodes efficiently in prisoners.

## Introduction

Higher levels of mental ill health have been found in prison populations compared to age, gender and education matched community samples with more than three-fold increases in all forms of depression and anxiety [1–3]. The high prevalence of depression and anxiety disorders in prisoners may be related to the characteristics of prisoners such as poorer cardiovascular and other physical health, alcohol and drug misuse, guilt, poor family support and experience of trauma and violence in childhood and adulthood [4–6]. The prison environment may also play a role in increasing depression and anxiety including lack of personal space, privacy, poor interpersonal relationships, social support, occupational roles and opportunities for exercise, occurrence of violence and coercion between prisoners, and lack of mental health access [3, 7].

The World Health Organisation [7] and The Bradley Report (2009) [8] recommended screening for mental disorder in the prison population to identify those who are a priority for clinical assessment by mental health professionals. Reasons for this include rising rates of suicide and self-harm [9], recidivism associated with poor mental health [10], and insufficient staff trained in mental health to meet the demand among prisoners [7, 10]. In England prisoners could be screened for depression and anxiety disorders alongside screening for risk of cardiovascular disease through NHS Healthchecks [11] to improve both mental and physical health together. However, self-rated screening instruments that are commonly used for the most prevalent mental disorders in prisoners and the community, namely depression and anxiety disorders, such as the Patient Health Questionnaire (PHQ-9) [12] for depression and the Generalised Anxiety Disorder Assessment (GAD-7) [13] might be susceptible to false positive results [14–16]. A meta-analysis of depression screening studies in prisoners estimated the prevalence of depressive episodes to be 19.1% (95% CI 15.0–23.9) but screening by self-rated measures of depression estimated depression to be present in 54.0% (95% CI 43.8–63.9%) [14]. There was considerable variation in the methods used to estimate depression by interview or self-rated measure, and the population varied by country, nature of prison, sentence or remand status, and selection by clinical or demographic factors. Previous research raises the possibility that symptoms commonly regarded as important for the diagnosis of depression might be commonly present among prisoners in the absence of depression such as difficulties with sleep in a noisy environment, unfamiliar food, guilt over crimes and low self-worth [3]. On the other hand there might also be underreporting of suicidal ideation so that prisoners avoid close observation and further restriction on their activities that is often the response of staff to reported suicidal ideation [17].

Therefore the validity of using community cut-off scores on the PHQ-9 and GAD-7 for possible caseness of moderate and severe depression and anxiety disorders of 10 or more and 15 or more merit further examination. Possible methods might include assessment for the presence of core symptoms of major depressive episode and generalised anxiety disorder are captured in prisoners scoring above caseness, whether there are expected associations with demographic and clinical factors such as alcohol use and cardiovascular health, and whether clusters of symptoms emerge in cases that are consistent or inconsistent with diagnostic criteria for major depressive episode and generalised anxiety disorder. There are also alternatives to detecting cases of depression and anxiety that can be utilised in relation to the PHQ-9 and GAD-7 such as varying cut-off scores, use of the PHQ-9 diagnostic algorithm [12] and examination of symptom profiles through cluster analysis.

Our objectives were to: 1. Estimate the proportions of prisoners over the age of 35 years who met threshold scores for possible moderate or severe depression or generalised anxiety on two commonly used screening measures, the PHQ-9 and GAD-7, or the diagnostic algorithm for the PHQ-9; 2. Explore how these thresholds relate to cardiovascular risk factors, previous alcohol intake, age group, ethnicity, deprivation, type of prison and sentence length; 3. To explore the validity of PHQ-9 and GAD-7 scores in relation to core symptoms of major depression and generalised anxiety disorder and clustering of items on these measures in prisoners scoring > 10 on the PHQ-9.

## Methods

A cross-sectional prevalence survey was conducted in prison healthcare services within the East Midlands [18]. In the period of data collection from September 2017 to January 2019, there were 13 male prisons in the region, with the healthcare services at six prisons agreeing to contribute to the research. These prisons varied in prison category, each housing prisoners of different sentence length.

The study drew on data collected at the time of the NHS Healthcheck [18]. All prisoners (regardless of sentence length or time served) who were deemed eligible for the NHS Healthcheck in Prisons were invited to participate (aged between 35 and 74 years old and with no exclusion diagnosis). Eligibility was sought using clinic reports from SystmOne, an NHS electronic medical record system, where those ineligible were subsequently filtered according to NHS Healthcheck eligibility criteria; those excluded were prisoners with established cardiovascular disease: coronary heart disease; strokes; transient ischaemic attacks; diabetes; atrial fibrillation; heart failure; peripheral artery disease and chronic kidney disease and those prisoners already taking statins. Each prison had a new report run every 2–3 weeks to allow for new prisoner receptions and to discount release or transferred prisoners from being invited to clinic.

At the clinic appointment, physical measures were collected from prisoners attending the NHS Healthcheck which included age, ethnicity, height, weight, body mass index (BMI), waist circumference, systolic and diastolic blood pressure (BP), smoking status, family history, alcohol intake using the Alcohol Use Disorders Identification Test (AUDIT) [19]. Physical activity was recorded using the General Practice Physical Activity Questionnaire (GPPAQ) [20]. Blood tests for creatinine, plasma glucose, lipids and HbA1c were requested. Age, ethnicity and last known home address were recorded. Index Multiple Deprivations (IMD) code was applied to the postcode of the prisoner’s last known home address; IMD 1 being the most deprived and IMD 10 the least [21]. A large percentage of prisoners were of no fixed abode (NFA) prior to incarceration and could not be assigned an IMD code. Sentence length was recorded where available.

### Measures of depression and anxiety

Once mental capacity had been assessed, written and oral Informed consent was obtained to complete the PHQ-9 and the GAD-7. The PHQ-9 and the GAD-7 are self-rated questionnaires that rate the frequency of symptoms 0 (not at all), to 3 (nearly every day) over the previous two-week period [12, 13] . Both classify total scores as none = 0–5, mild = 6–10, moderate = 11–15 and severe = ≥15. The PHQ-9 at > 10 has been found to have good sensitivity (88%) and specificity (88%) to detect major depressive disorder (MDD) [12]. The first two items (little interest or pleasure and feeling down, depressed or hopeless) are regarded as core symptoms of MDD [22]. The PHQ-9 can also be scored using the diagnostic algorithm [12] defining a case if one of the first two core symptoms are and five or more of the seven other items are also scored as present on more than half the days. The GAD-7 at > 10 has been found to have sensitivity of 89% and specificity of 82% for detection of generalised anxiety disorder [13]. The core symptoms of GAD are worry and these are asked in items 1 (feeling nervous, anxious or on edge), 2 (worry not stopping or controlling worry) and 3 (worry too much about different things) [13, 22]. Any participant scoring above therapeutic threshold (> 10) on the PHQ-9 and GAD-7 measures, was referred for clinical assessment to mental health services within the prisons.

### Statistical analysis

Summary measures were described using mean (standard deviation) or median (interquartile range) for continuous variables; categorical data were given as count (percentage). Means were compared using a two-sample t-test, medians using a two-sample Wilcoxon test and count data using a Chi-squared test. Correlations were calculated using Spearman’s rank correlation coefficient. Cronbach’s alpha was calculated for the PHQ-9 and GAD-7 [23]. Multinomial logistical regression models were fitted, due to the data failing the proportional odds assumption of a logistic regression model for PHQ-9 and GAD-7 separately. The model was fitted with the three categories of none/ mild, moderate and severe for both mental health measures, with moderate being used as the reference level. Age, prison, social deprivation level and sentence length were included in the model, with prison D being the reference level for the prison variable. Prison A was excluded from this part of the analysis because for logistic reasons they were only able to collect a small sample of data. Variables were removed if they were not statistically significant and the model was refitted until all variables were significant in at least one category.

A cluster analysis was performed to explore if there were one or more constructs contributing to a caseness score of 10 or more on the PHQ-9 and to examine symptom profiles of prisoners who had core symptoms of depression that might be used to identify which prisoners scoring 10 or more on the PHQ-9 merit further clinical investigation. For a subset of prisoners who scored > 10, the scores for individual PHQ-9 and GAD-7 questions were re-categorised into a score of 3 or any other score. A dendrogram was then plotted using agglomerative clustering and the optimal number of clusters was determined to be 5. All analyses were performed using R version 3.5.3 [24].

## Results

### Overall study population

Three thousand six hundred twenty prisoners were identified as eligible for a NHS Healthcheck over a 15-month period from September 2017 to January 2019. However, due to the throughput of prisoners entering and leaving the prison system and practical constraints, 1579 were invited to attend for a Healthcheck of whom 1207 (76.4%) consented to take part, with all but 2 prisoners completing the PHQ-9 and GAD-7 (total sample 1205, 76.3% response rate). The mean age (SD) of the study sample was 43.8 (7.8) years. Seven hundred fifty-two (62.4%) were either of no fixed abode or in the lowest quintile of the Index of Multiple Deprivation and 967 (81.2%) were white. Six hundred nineteen (51.4%) were on remand, 215 (17.6%) had less than 6 months of their sentence to serve and 371 (30.8%) were facing longer sentences.

Four hundred fifty-three (37.6%) of prisoners scored > 10 on the PHQ-9 and 378 (31.4%) > 10 on the GAD-7; 338 (27.1%) scored > 10 on both measures. Two hundred forty-nine (20.7%) and 217 (18.0%) prisoners scored > 15 on the PHQ-9 and GAD-7 respectively with 166 (13.8%) scoring > 15 on both measures. Using the diagnostic algorithm method, 216 (17.9%) prisoners were identified as having possible case level of depression. Cronbach’s alpha for the PHQ-9 was 0.87 and for the GAD-7 0.93.

### Sociodemographic factors

Table 1 shows that younger age was associated with increasing severity of depression on the PHQ-9 and anxiety on the GAD-7. In Table 2 multinomial logistic regression showed a 4% reduction in odds of being in the severe PHQ-9 category for each additional year of age compared with those in the moderate category (OR: 0.96 CI: 0.94–0.98 *P*:< 0.001). There was no evidence of an association between the moderate or none/mild PHQ-9 categories or for either comparison for GAD-7. There was no association between severity of depression or anxiety with either ethnicity or deprivation.

**Table 1:** Sociodemographic, sentence, prison, family history and QRISK2 variables with caseness for depression and anxiety

	PHQ-9				GAD-7			
	< 10 (*N* = 752)	≥10 to < 15 (*N* = 204)	≥15 (*N* = 249)	*p*-value	< 10 (*N* = 827)	≥10 to < 15 (*N* = 161)	≥15 (*N* = 217)	*p*-value
**Age** Mean (sd)	44.4(8.1)	43.6 (6.7)	42.2 (5.8)	< 0.001	45.9 (8.5)	42.7 (6.3)	43.1 (7.0)	< 0.001
Missing	0	0	0		0	0	0	
**Ethnicity**				0.608				0.251
White	604 (80.6)	171 (83.8)	212 (85.5)		673 (81.7)	131 (81.9)	183 (84.3)	
Black	31 (4.1)	8 (3.9)	7 (2.8)		29 (3.5)	11 (6.9)	6 (2.8)	
Asian	49 (6.5)	9 (4.4)	10 (4.0)		53 (6.4)	6 (3.8)	9 (4.2)	
Mixed/ Other	65 (8.7)	16 (7.5)	19 (7.7)		69 (8.4)	12 (7.5)	19 (8.8)	
Missing	3	0	1		3	1	0	
**Sentence Length (Years)**				0.046				0.005
< 1	142 (30.9)	33 (28.0)	51 (35.2)		143 (28.0)	47 (48.5)	37 (31.9)	
1–2	76 (16.5)	35 (29.7)	33 (22.8)		99 (19.4)	14 (14.4)	31 (26.7)	
2–3	53 (11.5)	12 (10.2)	11 (7.6)		61 (11.9)	6 (6.2)	9 (7.8)	
3–4	41 (8.9)	7 (5.9)	7 (4.8)		42 (8.2)	5 (5.2)	8 (6.9)	
4+	148 (32.2)	31 (26.3)	43 (29.7)		166 (32.5)	25 (25.8)	31 (26.7)	
Missing	292	86	104		316	64	101	
**IMD**				0.311				0.299
1–2	201 (27.4)	52 (26.5)	87 (35.7)		221 (27.5)	49 (31.4)	70 (32.9)	
3–4	123 (16.8)	34 (17.4)	49 (20.1)		136 (16.9)	25 (16.0)	45 (21.1)	
5–6	53 (7.2)	15 (7.7)	16 (6.6)		55 (6.8)	14 (9.0)	15 (7.0)	
7–8	62 (8.5)	17 (8.7)	15 (6.2)		67 (8.3)	9 (5.8)	18 (8.5)	
9–10	26 (3.6)	5 (2.6)	6 (2.5)		30 (3.7)	3 (1.9)	4 (1.9)	
No fixed address	268 (36.6)	73 (37.2)	71 (29.1)		295 (36.7)	56 (35.9)	61 (28.6)	
Missing	19	8	5		23	5	4	
**Prison**				< 0.001				< 0.001
A, life sentence	15 (2.0)	2 (1.0)	8 (3.2)		18 (2.2)	0 (0.0)	7 (3.2)	
B, remand prison	212 (28.2)	58 (28.4)	38 (15.3)		191 (23.1)	50 (31.1)	32 (14.8)	
C, medium term	74 (9.8)	11 (5.4)	11 (4.4)		76 (9.2)	10 (6.2)	9 (4.2)	
D, remand prison	163 (21.7)	43 (21.1)	105 (42.2)		218 (26.4)	63 (39.1)	107 (49.3)	
E, < 6 months to parole	103 (13.7)	53 (26.0)	59 (23.7)		122 (14.8)	20 (12.4)	35 (16.1)	
F, sex offenders	185 (24.6)	37 (18.1)	28 (11.2)		201 (24.3)	18 (11.2)	27 (12.4)	
Missing	0	0	0		0	0	0	
**Family History**				< 0.001				0.112
Yes	343 (48.8)	66 (35.1)	89 (39.2)		410 (53.4)	91 (61.1)	119 (59.2)	
No	360 (51.2)	122 (64.9)	138 (60.8)		358 (46.6)	58 (38.9)	82 (40.8)	
Missing	49	16	22		59	12	16	
**Smoking status**				< 0.001				< 0.001
Yes	593 (78.9)	27 (86.8)	19 (92.4)		658 (79.6)	143 (88.8)	199 (91.7)	
No	159 (21.1)	177 (13.2)	230 (7.6)		169 (20.4)	18 (11.2)	18 (8.3)	
Missing	0	0	0		0	0	0	
**Alcohol consumption Median (IQR)**	3 (0.0–8.0)	5 (0.0–13.0)	4 (0.0–18.3)	< 0.001	3 (0.0–8.3)	4 (0.0–17.0)	4 (0.0–19.3)	0.002
Missing	8	1	1		7	2	3	
**Height (M) Mean (sd)**	1.76 (0.1)	1.77 (0.1)	1.78 (0.1)	0.036	1.77 (0.1)	1.76 (0.1)	1.78 (0.1)	0.197
Missing	5	2	2		6	2	1	
**Weight (kg) Mean (sd)**	84.37 (15.3)	84.40 (17.2)	81.87 (16.2)	0.048	84.60 (15.5)	82.49 (16.0)	82.03 (16.7)	0.018
Missing	2	2	0		4	0	0	
**BMI**				0.046				0.045
**< 25**	275 (36.6)	83 (41.1)	116 (47.0)		303 (36.8)	67 (41.9)	104 (48.1)	
**≥25 < 30**	304 (40.5)	70 (34.7)	84 (34.0)		328 (39.8)	59 (36.9)	71 (32.9)	
**≥30**	172 (22.9)	49 (24.3)	47 (19.0)		193 (23.4)	34 (21.2)	41 (19.0)	
Missing	1	2	2		3	1	1	
**Waist (cm) Mean (sd)**	93.71 (12.0)	94.42 (13.0)	91.20 (11.6)	0.020	94.23 (12.0)	91.00 (11.5)	91.78 (12.0)	0.002
Missing	59	15	10		65	8	11	
**QRISK2 Score**				0.015				0.008
< 10%	668 (88.8)	177 (86.8)	235 (94.4)		730 (88.3)	143 (88.8)	207 (95.4)	
≥10%	84 (11.2)	27 (13.3)	14 (5.6)		97 (11.70)	18 (11.2)	10 (4.6)	
Missing	0	0	0		0	0	0	
**Exercise status**				< 0.001				< 0.001
Active	318 (44.6)	74 (38.0)	56 (23.1)		346 (44.2)	46 (29.7)	56 (26.3)	
Moderately active	132 (18.5)	31 (15.9)	39 (16.1)		139 (17.8)	28 (18.1)	35 (16.4)	
Moderately inactive	93 (13.0)	22 (11.3)	38 (15.6)		101 (12.9)	19 (12.3)	33 (15.5)	
Inactive	170 (23.8)	68 (34.9)	110 (45.3)		197 (25.2)	62 (40.0)	89 (41.8)	
Missing	39	9	6		20	6	28	
**Diastolic blood pressure (mmHg) Mean (sd)**	77.60 (11.6)	78.19 (10.8)	78.18 (11.6)	0.428	77.87 (11.6)	77.62 (11.1)	77.79 (11.2)	0.883
Missing	0	0	0		0	0	0	
**Systolic blood pressure (mmHg) Mean (sd)**	126.40 (15.1)	125.45 (14.8)	124.14 (16.8)	0.043	126.05 (15.3)	126.66 (15.0)	124.08 (16.1)	0.157
Missing	1	0	0		1	0	0	
**Serum creatinine (umol/L) Mean (sd)**	80.95 (12.5)	80.38 (11.6)	77.74 (12.2)	0.026	80.64 (12.3)	80.70 (12.9)	77.98 (12.0)	0.100
Missing	426	125	145		456	107	133	
**HbA1c mmol/mol Mean (sd)**	37.06 (8.4)	37.84 (9.0)	36.13 (4.6)	0.607	37.14 (8.3)	38.17 (9.8)	35.65 (4.5)	0.356
Missing	489	137	186		196	121	497	
**HDL cholesterol (mmol/L) Mean (sd)**	1.20 (0.3)	1.16 (0.3)	1.17 (0.3)	0.380	1.19 (0.3)	1.19 (0.4)	1.16 (0.2)	0.545
Missing	454	126	164		486	114	144	
**Plasma glucose level (mmol/L) Mean (sd)**	5.04 (1.0)	5.16 (0.8)	5.12 (0.7)	0.508	5.02 (0.7)	5.50 (1.9)	5.06 (0.8)	0.464
Missing	612	176	199		669	140	178	

PHQ-9 < 10 = no/mild depression, > 10 to < 15 = moderate depression, > 15 moderately severe or severe depression; GAD-7 < 10 = no/mild/moderate anxiety, > 10 to < 15 = moderately severe anxiety, > 15 = severe anxiety

**Table 2:** Predictors of no/mild caseness and moderately severe caseness on PHQ-9 with moderate caseness as reference

Predictor	Total PHQ-9 (< 10)		Total PHQ_9 (≥ 15)	
	Odds ratio (95% CI)	***P***-value	Odds ratio (95% CI)	***P***-value
**Age** (years)	0.99 (0.97–1.01)	0.418	0.96 (0.94–0.98)	< 0.001
**Prison** (reference remand prison D)				
B, remand prison	1.07 (0.69–1.68)	0.753	0.28 (0.18–0.44)	< 0.001
C, medium term	0.55 (0.27–1.13)	0.102	0.23 (0.12–0.46)	< 0.001
E, near parole	1.99 (1.24–3.20)	0.004	0.87 (0.57–1.31)	0.492
F, sex offender	0.79 (0.48–1.30)	0.362	0.28 (0.17–0.44)	< 0.001
**Alcohol consumption** (high)	1.63 (1.15–2.30)	0.005	1.72 (1.24–2.39)	0.001

B, C, D, E and F refer to prison sites

### Prison and sentence factors

Sentence length was significantly associated with both PHQ-9 and GAD-7, with the percentage of prisoners with a sentence < 2 years being higher in the severe categories of depression and anxiety compared to the other categories (Table 1). Severity of PHQ-9 and GAD-7 also showed significant variability in prevalence across different prison types with higher scores in local remand prisons and prisons where prisoners had less than 6 months to serve. One local remand prison had very high rates of severe depression on the PHQ-9, and very high rates of both moderate and severe anxiety on the GAD-7.

### Cardiovascular risk

Table 1 shows that overall severity of depression and anxiety as measured by the PHQ-9 and GAD-7 were inversely associated with higher cardiovascular risk score on QRISK2 [25] classified as a 10-year risk > 10% [26]. However, a more variable pattern emerges when individual risk factors are examined. Higher alcohol consumption, smoking and inactivity in relation to exercise were associated with higher PHQ-9 and GAD-7 scores. Family history of cardiovascular disease, higher serum creatinine and higher systolic blood pressure were less likely to be associated with higher PHQ-9 scores but there was no association with GAD-7 scores. Higher body mass index and waist circumference were inversely associated with severity on both the PHQ-9 and GAD-7. Diastolic blood pressure, Hb1Ac, HDL cholesterol and plasma glucose were not associated with either PHQ-9 or GAD-7 score.

### Predictors of non-caseness and severe caseness on the PHQ-9 and GAD-7

Using a multinominal logistic regression analysis, Table 2 demonstrates that high alcohol consumption and being in a prison catering for prisoners near parole or release were associated with no or mild depression (PHQ-9 0–9) rather than moderate depression (PHQ-9 > 10). Younger age, prison type and high alcohol consumption were all associated with severe depression (PHQ-9 > 15) rather than moderate depression (PHQ-9 10–14). In the multinominal logistic regression analysis, none of the sociodemographic, prison and sentence, and cardiovascular risk factors were associated with any level of caseness on the GAD-7.

### Distribution of items on PHQ-9 and GAD-7

Of those scoring > 10 on the GAD-7, 283 (74.9%) of 378 scored 3 on any of the three core items for worry (Items 1, 2 and 3). When examining those who scored > 15 on the GAD-7, 209 (96.3%) of 217 scored a 3 on at least one of the core symptoms of worry.

Of those who scoring > 10 on the PHQ-9, only 232 (51.2%) of 453 participants scored a 3 (‘nearly every day’) on either of the core symptoms of depression (‘little interest or pleasure in doing things’ or ‘feeling down, depressed or hopeless’). When examining those who scored > 15 (severe), only 185 (74.3%) of 249 scored 3 on either of these two items.

**Fig. 1:**
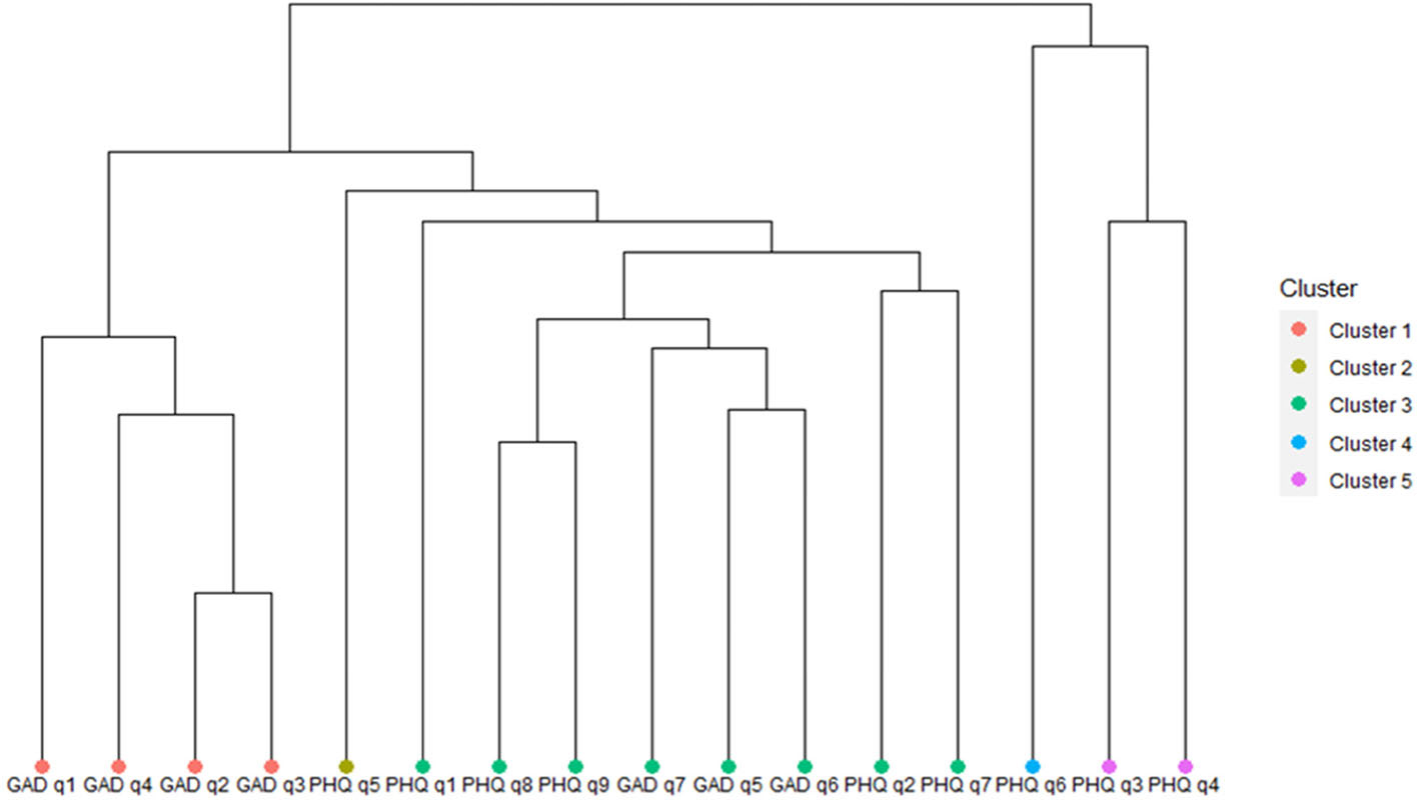
Dendrogram of items scoring 3 on PHQ-9 and GAD-7 in prisoners scoring 10 or more on PHQ-9

Figure 1 shows a dendogram of prisoners scoring > 10 on the PHQ-9 using all the items of the PHQ-9 and GAD-7. It shows the similarity of responses and clustering of items from the PHQ-9 and GAD-7 that scored 3. Cluster 1 (probable generalised anxiety disorder) includes GAD-7 items 1 (nervous/anxious/on edge), 4 (trouble relaxing), 2 (unable to control worry) and 3 (worrying too much). All the main constructs of GAD (items 1, 2 and 3) are contained within the same cluster. Cluster 2 (poor appetite) contains PHQ-9 item 5 (altered appetite) which is an outlier. Cluster 3 (probable depressive episode) was the largest cluster and included PHQ-9 items 1 (little interest/pleasure), 8 (moving or thinking slowly or fidgety/restless), 9 (suicidality), 2 (down/ depressed/hopeless) and 7 (poor concentration) along with GAD-7 items 5 (feeling restless), 6 (annoyed/ irritated) and 7 (afraid something awful will happen). Cluster 4 (guilt and low self-worth) contained PHQ-9 item 6 (guilt and low self-worth) which is the most dissimilar to the other items and an outlier. Cluster 5 (insomnia and fatigue) included PHQ-9 items 3 (poor sleep) and 4 (tired/no energy). Therefore, cluster 3 may identify people with probable depression disorder in prisoners scoring 10 or more on the PHQ-9.

## Discussion

We found a high prevalence of prisoners scoring above conventional thresholds for moderate and severe depression and anxiety on the PHQ-9 and GAD-7. However, around half of prisoners with moderate depression on the PHQ-9 did not exhibit core symptoms of depression on a daily basis, and a quarter of prisoners with moderate anxiety on the GAD-7 did not have daily core symptoms of worry. Ninety-six per cent of prisoners with severe anxiety on the GAD-7 had daily core symptoms of anxiety suggesting that a GAD-7 score > 15 has high specificity for the presence of generalised anxiety disorder. However, one quarter of prisoners with severe depression on the PHQ-9 did not have daily core symptoms of depression. Increasing threshold scores of caseness on screening measures for anxiety and depression [27] from > 10 to > 15 on the GAD-7 would provide a high specificity for generalised anxiety disorder but not on the PHQ-9 for depression. Given the possibility of under-reporting as well as over reporting of some symptoms such as suicidality, a caseness threshold of 10 or more may be satisfactory for the GAD-7 but not for the PHQ-9. The diagnostic algorithm suggested that only 17.9% prisoners had a depressive episode which is in the expected range for caseness for a depressive episode (15.0–23.9%) when standardised psychiatric interviews were used in previous studies [14]. However, the diagnostic algorithm method tends to have low sensitivity [28] for depressive episodes so it may miss some cases, including some who may be at risk of suicide and self-harm. Therefore consideration might be given to other methods of utilising the PHQ-9 and GAD-7 since both the PHQ-9 and GAD-7 showed excellent internal consistency [23] in this sample of prisoners and their completion was highly acceptable to prisoners. The problems of case detection on the PHQ-9 using the community caseness threshold of 10 is not due to the low internal reliability of these measures among prisoners.

We found many univariate associations between no/mild and moderate case levels of depression and anxiety and younger age, shorter and longer sentence length, prison, higher alcohol consumption and lower cardiovascular risk. Many of these cardiovascular risk and prison or sentence factors were strongly related to age. Depression and anxiety caseness were not independent of each other either with 27.1% of the sample scoring 10 or more on both the PHQ-9 and GAD-7. As a result in the multinominal logistic regression analysis only prison and alcohol consumption were associated with caseness thresholds of > 10 and > 15 on the PHQ-9 and additionally young age was associated with the caseness threshold of > 15 on the PHQ-9. The relationship between alcohol consumption and PHQ-9 score was complex. High alcohol consumption was associated with severe depression but also with no or mild depression compared to moderate depression. Alcohol lowers mood and not being exposed to it might lift people out of milder states of depression but not out of severe depression [29]. No variables were associated with caseness thresholds of > 10 or > 15 on the GAD-7. However, the relationship of anxiety symptoms to demographic factors, alcohol use, cardiovascular disorders, sentencing and prisoners have been much less explored than for depression in prisoners or other samples. Therefore, the lack of an association between these variables and the severity of anxiety in this sample is open to interpretation. The lack of an association between the severity of depression and factors such as social deprivation and higher cardiovascular risk was not expected, possibly because many prisoners were of no fixed abode so not contributing to assessment of social deprivation, and the sample was predominantly white British in ethnicity [18].

One of the two remand prisons and a near parole prison for prisoners with only 3–6 months left to serve of a longer sentence showed increased rates of caseness on the PHQ-9 compared to the other prisons, including another remand prison. These prisons and the other remand prison with lower rates of caseness on the PHQ-9 had high ‘churn’ (turnover) rates, with prisoners moving through these establishments at high rates. Precision in relation to the denominator mean that estimates of prevalence of caseness is less certain in prisons with high churn, but the churn also means less opportunity to establish social support which has been related to common mental disorder in prisoners [3]. Both remand and sentenced prisoners report high rates of victimisation in prison and low rates of social support available to them in prison and the community [3]. The remand prison with high PHQ-9 caseness was a large prison associated with relative overcrowding compared to the other remand prison with lower caseness of depression. There were also high rates of caseness on the PHQ-9 and GAD-7 among a small sample of only 25 prisoners with life sentences but this was too small a sample to draw firm conclusions.

Given previous concerns about false positives when screening prisoners for mental health problems with community derived thresholds [14–16], we conducted a dendrogram of scores of 3 or more on the core items of the PHQ-9 and GAD-7 in all those who scored > 10 on the PHQ-9. We found five clusters, the largest of which (cluster 3) had daily core symptoms of depression with suicide ideation, poor concentration, feeling slowed down or restless, irritability or fear of something bad happening. This cluster might consist of prisoners with symptoms suggestive of a depressive disorder meriting further clinical examination. In relation to the other clusters, cluster 1 contained prisoners scored highly on the first four worry and related symptoms of GAD-7, including the three core symptoms, but not on the remaining GAD-7 items or PHQ-9 items. A previous study indicated that over 40% of male sentenced and nearly 60% of remand prisoners reported frequent worry [3]. Cluster 2 related to Item 5 (altered appetite) reflecting a prison diet that is rather different from their previous diet. Cluster 4 was an outlier consisting of worthlessness and guilt (item 6 on the PHQ-9) which was frequently scored highly (25.1%). Guilt may have been associated with the crime committed or letting down family while low self-esteem may have been prominent throughout their lives, not necessarily related to current feelings [30, 31]. Cluster 5 consisted of high scores on poor sleep and fatigue; insomnia and fatigue are frequently reported by prisoners in noisy environments [3].. Thus there are possible environmental, guilt and stress factors in a prison setting influencing the scoring of a number of items on both the PHQ-9 (e.g. items 3–6) and GAD-7 (items 1–4), potentially inflating PHQ-9 scores, and GAD-7 scores to a lesser extent, in a prison setting.

We have confirmed previous suspicions that using conventional community cut-off scores on self-rated measures of depression tends to overdiagnose depression. Using a threshold of 10 or more on the PHQ-9 to detect depression caseness seems to overinflate detection rates because it captures too many prisoners struggling with distress from poor sleep and fatigue, poor appetite, guilt or low self-worth who do not have core symptoms of a depressive episode nearly every day over the previous 2 weeks as defined by DSM-V [22]. Although these problems have been documented as distressing to prisoners before [3], this study is the first to provide evidence that such problems may be contributing to an overdiagnsosis of depressive episodes using a community threshold of caseness for depression of 10 or more on the PHQ-9. A positive score on the PHQ-9 diagnostic algorithm, a cut-off score of 15 or more on the PHQ-9, or a symptom profile similar to cluster 3 on the PHQ-9 and GAD-7 in prisoners scoring 10 or more on the PHQ-9 are worth exploring singly and in combination as a method of efficiently finding prisoners who may have a depressive episodes in further research using a gold standard psychiatric interview. However, it is worth noting that one study which used multiple self-rated questionnaires only improved the specificity of detection of depression by a marginal amount [15].

Caseness for depression or anxiety disorder would result in a mental health assessment by a qualified mental health professional followed by an appropriate treatment plan. The latter might involve medication, psychological treatment or a change to social or environmental factors within the prison depending on the nature of the mental health problem and factors contributing to its maintenance.

### Strengths and limitations of the study

Strengths of the study were the large sample size, the sampling of prisoners of different sentence duration in different types of prison, care to establish complete denominators of eligible prisoners in each prison, including those with high turnover, and systematic collection of a broad range of cardiovascular risk and lifestyle factors alongside screening measures of depression and anxiety. We used standardised operating procedures for NHS Health check assessment and validated tools for assessment of mental health.

The study recruited participants eligible for an NHS Healthcheck. Prisoners with existing cardiovascular disease or with established risk factors for cardiovascular disease, such as diabetes mellitus and hypertension, were excluded. These included many non-white prisoners who had high rates of cardiovascular disorder and were not included in this sample, thereby weakening any association between ethnicity and possible caseness for depression and anxiety. Around 70% of the sample were of no fixed abode or in the bottom two quintiles of the IMD, lessening the chances of demonstrating an association between deprivation and anxiety or depression caseness.

Overall, 76% of the eligible population who were invited took part. There were only minor demographic differences in those who took part and those who did not. However, qualitative work from our study with attending and non-attending prisoners and prison staff highlighted that stigma and crowded waiting areas may have deterred some prisoners from participating [17]. Both these factors might be more of a barrier in prisoners with anxiety and depression compared to other prisoners. Furthermore, some PHQ-9 scores may have been underscored since some prisoners expressed reluctance to endorse the PHQ-9 item on suicidality because they did not want to be monitored more closely.

Those attending the NHS Healthcheck assessment were more likely to have a family history of cardiovascular disease than other prisoners. However, moderate or severe anxiety or depression were not associated with attendance despite previous reports of increased somatic symptoms with anxiety or depression in prisoners [3]. Therefore, possible response bias due to a positive family history of cardiovascular disease probably did not affect caseness or associations of risk factors with anxiety or depression caseness thresholds.

A limitation of the study was that there was no standardised diagnostic interview to compare with thresholds on the PHQ-9 and GAD-7, the diagnostic algorithm using the PHQ-9 and to confirm or refute that the symptom cluster 3 among PHQ-9 scorers > 10 was associated with a high specificity for depressive disorder. Further research should be carried out first before altered thresholds on the PHQ-9 or GAD-7, the diagnostic algorithm for the PHQ-9 or symptom profiles are used singly or in combination in clinical practice to define which prisoners should have further mental health assessment. A standardised psychiatric interview is also required to diagnose other types of mental disorders found in prisoners e.g. serious mental illness, personality disorder, post-traumatic disorder or substance use disorder [1, 32]. The results may not generalise to female, under 35-year prison populations or prison populations in low- and middle-income countries.

The study was cross-sectional and not prospective in design so the associations between depression caseness with young age, type and nature of prison, and high alcohol consumption might relate to stressful periods of transition from the community to prison or vice-versa, or due to persistent mental ill-health. One study followed a cohort of prisoners over 9 weeks suggest some decline in symptoms in some male prisoners with depression but not in remand prisoners [32].

## Conclusion

PHQ-9 and GAD-7 scores may be inflated in prisoners by environmental, cognition and stress related factors. Further research using standardised diagnostic interviews should explore whether the use of higher threshold scores, diagnostic algorithm on the PHQ-9 and the pattern of items on the PHQ-9 and GAD-7 among prisoners scoring 10 or more among high scorers singly or in combination efficiently identifies cases of depressive episode and generalised anxiety disorder that merit further clinical examination by a mental health professional.

## Data Availability

Deidentified participant data collected in the study and a data dictionary will be made available to others after publication by direct e-mail to the corresponding author. Each application will be considered on a case by case basis by the authors.

## Abbreviations

AUDIT: Alcohol Use Disorders Identification Test
BP: Blood pressure
BMI: Body mass index
CI: Confidence interval
GAD-7: Generalised Anxiety Disorder Assessment
GPPAQ: General Practice Physical Activity Questionnaire
IMD: Index multiple Deprivations
MDD: Major depressive disorder
NFA: No fixed abode
NHS: National Health Service
PHQ-9: Patient Health Questionnaire
SD: Standard deviation
